# Male relatedness and familiarity are required to modulate male-induced harm to females in *Drosophila*

**DOI:** 10.1098/rspb.2017.0441

**Published:** 2017-08-09

**Authors:** Sally Le Page, Irem Sepil, Ewan Flintham, Tommaso Pizzari, Pau Carazo, Stuart Wigby

**Affiliations:** 1 Edward Grey Institute, Department of Zoology, University of Oxford, Oxford, UK; 2 Behaviour, Ecology and Evolution group, Instituto Cavanilles of Biodiversity and Evolutionary Biology, University of Valencia, Valencia, Spain

**Keywords:** sexual conflict, sexual selection, kin selection, inclusive fitness, social behaviour, *Drosophila*

## Abstract

Males compete over mating and fertilization, and often harm females in the process. Inclusive fitness theory predicts that increasing relatedness within groups of males may relax competition and discourage male harm of females as males gain indirect benefits. Recent studies in *Drosophila melanogaster* are consistent with these predictions, and have found that within-group male relatedness increases female fitness, though others have found no effects. Importantly, these studies did not fully disentangle male genetic relatedness from larval familiarity, so the extent to which modulation of harm to females is explained by male familiarity remains unclear. Here we performed a fully factorial design, isolating the effects of male relatedness and larval familiarity on female harm. While we found no differences in male courtship or aggression, there was a significant interaction between male genetic relatedness and familiarity on female reproduction and survival. Relatedness among males increased female lifespan, reproductive lifespan and overall reproductive success, but only when males were familiar. By showing that both male relatedness and larval familiarity are required to modulate female harm, these findings reconcile previous studies, shedding light on the potential role of indirect fitness effects on sexual conflict and the mechanisms underpinning kin recognition in fly populations.

## 1. Introduction

The evolutionary strategies that maximize female fitness may simultaneously hamper male fitness and *vice versa*, generating sexual conflict over reproductive decisions [1–3]. This conflict often arises because intense competition among males over access to mating and fertilization opportunities can harm females (i.e. reduce their fitness). Such harm has been likened to a tragedy of the commons [4–7], in which selfish exploitation results in the depletion, or even destruction, of a shared resource. Male harm of females may occur through a number of pathways including sexual harassment, sexual coercion, traumatic insemination, male accessory gland products, pathological polyspermy and infanticide [2]. In all these cases, sexual selection promotes male strategies even if they happen to harm females in the process (i.e. collateral female harm), or precisely because they harm females (e.g. [8]). Male harm of females is emerging as an important factor in population ecology and evolution, as increasing evidence indicates its role in a number of fundamental processes, such as dispersal [9], population extinction [10] and intersexual coevolution [2]. However, the mechanisms underpinning the variation in the severity of female harm observed across species and populations remain little understood.

Recent theoretical work has suggested that indirect fitness effects might play a key role in modulating male harm to females [6,11–14]. This happens whenever males tend to compete with males to whom they are more genetically related than the population average, for example when population viscosity limits dispersal and competition is not exclusively local [14,15]. In this context, a male may indirectly reduce his own inclusive fitness by harming females that could also reproduce with his male relatives, and this is expected to relax male–male competition and selection for male traits that harm females [6,11–14].

While the expectation that, under some circumstances, within-group male relatedness reduces the intensity of intra sexual competition has received empirical support (e.g. [16–19]), the notion that within-group male relatedness might also reduce female harm is only beginning to be investigated. Consistent with this idea, female least killifish, *Heterandria formosa*, died younger and produced progressively smaller offspring when experimentally mated to males that are unrelated to each other, compared with females mated to males highly related to each other (but always unrelated to the female; [20]). Similarly, female bulb mites, *Rhizoglyphus robini*, laid more eggs over a 2-day period when paired for 5 days with males that had experimentally evolved in populations comprising their full siblings than when paired with stock males [21].

The influence of male relatedness on female harm has also been explored in the fruit fly, *Drosophila melanogaster*. Carazo *et al.* [22] found that females had higher lifetime reproductive success and slower reproductive ageing (a more gradual decline in fecundity and fertility with age) when exposed to a triplet of brothers that were unrelated to the female but had been raised together as larvae than when exposed to a triplet of males that were unrelated to each other and had been raised separately as larvae. These patterns have now been explored by different research groups and in different *D. melanogaster* populations [23–26] resulting in some studies reporting results consistent with Carazo *et al*.'s findings and others reporting no effects (summarized in electronic supplementary material, table S1), suggesting that these effects are not entirely consistent and that they might be modulated by other mechanisms.

One such mechanism might be familiarity. Hollis *et al.* [24] identified larval familiarity among males as a requirement for reduced harm to females. By introducing a new treatment in which females were exposed to males that were related to each other but raised apart as larvae, this study showed that males were only benign to females when they were related and raised together as larvae. These results are consistent with larval familiarity acting as a kin recognition mechanism, as demonstrated in other taxa [27–32]. In principle, these results may also indicate that male flies might have evolved to reduce female harm strategically in response to male familiarity *per se*, independently of relatedness, through direct (rather than indirect) fitness effects [24]. For example, mechanisms such as reciprocity might reduce competition among familiar males, and this may in turn reduce female harm.

A scenario in which variation in male harm is entirely predicted by relatedness, not familiarity, would suggest that flies use genetic cues to recognize kin and reduce harm in the presence of relatives to gain indirect fitness benefits. A scenario in which variation in male harm is entirely predicted by male familiarity, not relatedness, would be consistent both with the idea that direct benefits associated with familiarity drive changes in female harm, and the idea that female harm is driven by indirect effects, whereby flies may rely entirely on familiarity cues to recognize kin. Finally, variation in male harm may be predicted by the interaction between relatedness and familiarity cues. For example, indirect fitness effects may reduce male harm to females when males are related, but male flies may only be able to recognize relatives under familiarity [33]. However, because no study has tested the fully factorial combination of relatedness and familiarity [22–26], the relative roles of these factors remain unresolved.

In this study, we conducted an experiment using a novel, fully factorial design to isolate the separate effects of relatedness, familiarity (shared larval environment) and their interaction on male sexual behaviour (as measured through assays of male–male aggression, courtship and mating rates) and female harm (as measured through female lifetime reproductive success, reproductive ageing, lifespan and reproductive lifespan) in *D. melanogaster*. We used four different social environments in which males were: (i) related and familiar, (ii) related and unfamiliar, (iii) unrelated and familiar and (iv) unrelated and unfamiliar. While we found no effect on male behaviours, we did observe an interaction between male relatedness and larval familiarity, thereby showing that larval familiarity alone is insufficient to reduce harm to females. Male relatedness increased female reproductive success, lifespan and reproductive lifespan, and slowed reproductive ageing, but only when males were familiar.

## 2. Methods

### (a) Stock cultures

We used a laboratory-adapted, wild-type Dahomey stock of *Drosophila melanogaster*, maintained in large, outbred populations since 1970 [34,35] at 25°C in a non-humidified room and a 12 : 12 h light : dark cycle. This is the same stock used by Carazo *et al.* [22,23]. All flies were maintained in cages containing bottles of Lewis medium [36] with overlapping generations.

### (b) Male treatments

**Figure 1.**
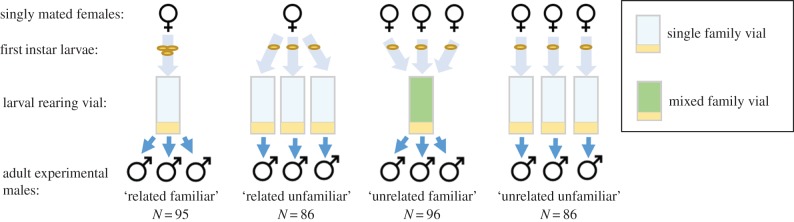
Scheme of how we generated the four male treatments. Each rearing vial contained 15 larvae, either all 15 from one singly mated female (single family vial) or one larva from each of 15 singly mated females (mixed family vial). We collected adult virgin males from these rearing vials, which were immediately housed in their experimental triplets: ‘related familiar’, ‘related unfamiliar’, ‘unrelated familiar’ or ‘unrelated unfamiliar’.

We produced triplets of males belonging to one of four treatments generated from a fully factorial cross of relatedness and familiarity in the larval environment: related and familiar, related and unfamiliar, unrelated and familiar, and unrelated and unfamiliar (figure 1).

To generate each experimental male triplet, we created families using parents that were 2 days post-eclosion, and had been collected as eggs from the stock population and reared at standard larval density at 25°C [34]. We paired a single virgin male and female for 12 h in individual larval collection chambers containing a Petri-dish filled with hard grape agar (550 ml water, 25 g agar, 300 ml grape juice concentrate and 21.25 ml 10%w/v Nipagin) with a smear of live yeast paste, before discarding the males. Twenty-four to thirty-six hours after egg laying, we picked larvae with a mounted needle into 36 ml vials containing 8 ml of Lewis medium, collecting 60 larvae in total per family over a period of 3 days. Any families that failed to produce 60 larvae were excluded.

From each of 135 families, 45 larvae were divided equally among three ‘single family’ vials and 15 larvae were distributed individually among each of 15 ‘mixed family’ vials. Thus, each ‘single family’ vial contained 15 larvae from a single family, and each ‘mixed family’ vial contained 15 larvae from 15 randomly allocated families (figure 1). These vials were kept at 18°C and adult virgin males were collected within 16 h of eclosion.

Virgin males were immediately aspirated and housed in vials of Lewis medium and excess live yeast grains at 18°C in their experimental triplets: ‘related familiar’, ‘related unfamiliar’, ‘unrelated familiar’ and ‘unrelated unfamiliar’. ‘Related familiar’ comprises three males collected from the same ‘single family’ vial. ‘Related unfamiliar’ comprises one male taken from each of the three ‘single family’ vials of the same family. ‘Unrelated familiar’ comprises three males taken from the same ‘mixed family’ vial. ‘Unrelated unfamiliar’ comprises one male taken from each of three ‘single family’ vials of three different families. No family contributed to more than one vial of each related familiar and related unfamiliar treatments, and families were randomly assigned so that each had an equal contribution to the unrelated familiar and unrelated unfamiliar treatments. Two days before the introduction of females, males were transferred to fresh vials and kept at 25°C. To produce virgin females, we reared eggs from the cage population at 18°C at standard density (approx. 250 flies per 75 ml bottle containing 45 ml of Lewis medium) in parallel with male collection, collected adult females under ice anaesthesia and aged them at 25°C in individual yeasted vials for 3 days before the start of the experiment.

We performed the experiment across two blocks, producing a combined total of 95 related familiar triplets (39 in block 1, 56 in block 2), 86 related unfamiliar triplets (37 in block 1, 49 in block 2), 96 unrelated familiar triplets (22 in block 1, 74 in block 2) and 86 unrelated unfamiliar triplets (33 in block 1, 53 in block 2). Differences in sample sizes across treatments are due to some flies escaping and stochastic variation in the number of adult males emerging in each family vial within the short collection period.

### (c) Behavioural observations

On day 1, we added a single virgin female to each male triplet in a randomly numbered vial to blind the observer to the treatment throughout data collection. On days 2, 3, 4, 5, 8, 9, 10, 11 and 12, we observed the vials during eight scans in the morning (only seven scans on day 2, block 1), 10–20 min apart and recorded whether any males were displaying aggression [37], courtship [38] and mating behaviours. Note that in Carazo *et al.* [22], triplets of males were replaced at regular intervals to prevent males co-ageing with the female. The set-up we used to generate unrelated familiar males prevented us from replacing males during the experiment, therefore males were allowed to age with the female in this study, and as such, we did not expect a similarly strong level of female harm as reported in [22].

Flies were transferred to fresh vials under light CO_2_ anaesthesia on days 3, 5, 8 and 11 in both blocks and additionally on day 15 in block 2, and the vials were retained to collect adult offspring (see Fitness measures). Vials were discontinued upon the female's death, and we recorded the day of death up to day 15 in block 1 and up to day 19 in block 2 after which time any remaining females (6% across both blocks) were censored. We also censored vials in the event of male death (four related familiar vials, one related unfamiliar vial, one unrelated familiar vial, one unrelated unfamiliar vial) or flies escaping during handling (one related familiar vial, one related unfamiliar vial).

### (d) Fitness measures

Vials containing the offspring of experimental groups were reared at 25°C for 16 days, allowing sufficient time for offspring to develop to the pupal or adult stage, when they were then frozen. To account for different egg-to-adult development times between vials, we counted adult flies and pupae that had reached the P13 phanerocephalic pupal phase [39], identified by the black wing colour, and included both in offspring counts.

### (e) Statistical analysis

Survival models were performed using JMP [40]. All other models were performed using the MASS package [41] in R [42] using type III sums of squares to calculate *p*-values. For all analyses, we included block—and all interaction terms that include block—as fixed effects. In all cases, the interaction terms that include block were not significant (electronic supplementary material, table S2), so we removed these terms from the models and kept block as a fixed main effect. While we know which families contributed to the related familiar, related unfamiliar and unrelated unfamiliar treatments, our experimental design makes it impossible to know the family identities of flies in the unrelated familiar treatment. As our knowledge of family identity is confounded with treatment, we were not able to include family identity in the full model analysis.

For aggression and courtship, we analysed the number of scans per day in which the behaviour was observed with a binomial penalized quasi-likelihood GLMM [43], with relatedness, familiarity and their interaction, and block as fixed effects, and day within vial ID as a nested random effect. For mating rate, we analysed whether or not a mating was observed for each day using a binomial penalized quasi-likelihood GLMM with relatedness, familiarity and their interaction, and block as fixed effects, and vial as a nested random effect.

For female reproductive success, we analysed the total number of offspring produced during the experiment. Only 27 of the 357 females were still reproducing at the end of the experiment, and short-term reproductive success is known to be a strong predictor of long-term reproductive success in this species [44]. Therefore, our measurements of reproductive success during the experiment can be considered a very close estimate of lifetime reproductive success. Vials in which a male died before the death of the female were excluded from this analysis. We analysed lifetime reproductive success with a quasi-Poisson GLM with relatedness, familiarity and their interaction, and block as fixed effects. For female reproductive ageing, we divided the number of offspring laid in each time period by the number of days in that period to create an estimated daily offspring measure that accounts for the differing number of days in each time period. Vials in which a male died before the death of the female were right-censored in this analysis. We analysed daily offspring estimates with a Poisson penalized quasi-likelihood GLMM with relatedness, familiarity and day and their interactions, and block as fixed effects, and the vial ID as a random effect.

To estimate female reproductive lifespan, we used the last day of the last time period in which the female reproduced. We fitted proportional hazards models for female lifespan and female reproductive lifespan, with relatedness, familiarity and their interaction, and block as fixed effects. Vials were right-censored in the analysis when male death, male escape or the end of the experiment preceded female death or the end of reproduction.

## 3. Results

### (a) Male behaviour

The frequency of male–male aggression, courtship and mating were not significantly affected by male relatedness, larval environmental familiarity or their interaction (electronic supplementary material, tables S3 and S4).

### (b) Female harm

**Figure 2.**
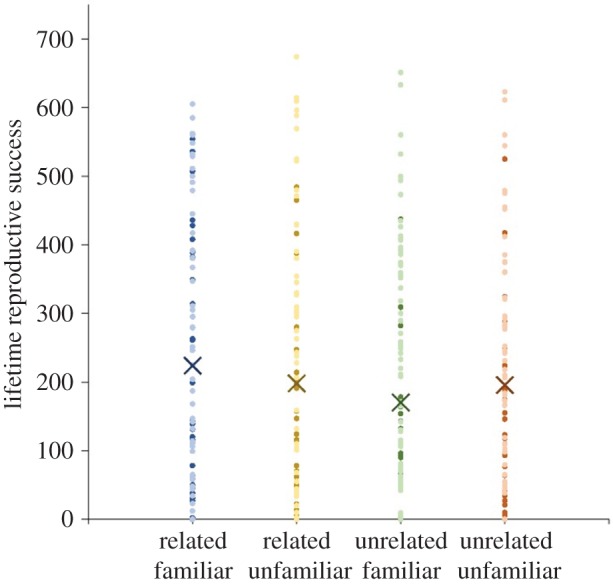
The effect of male relatedness and larval familiarity on female lifetime reproductive success. Points show the total number of offspring laid by each female during the experimental period that reached adult and P13 pupal stage from the first experimental block (dark points), the second experimental block (light points) and predictions from the generalized linear mixed model (crosses). Females mated to triplets of males that were related produced significantly more offspring than those mated to triplets of unrelated males (*p* < 0.05). There was no difference in lifetime reproductive success between females mated to triplets of familiar and unfamiliar males (*p* > 0.05).

Female lifetime reproductive success was significantly increased by relatedness among male triplets (*t*_349_ = −2.1 *p* = 0.034), but there was no significant effect of familiarity (*t*_349_ = −0.97, *p* = 0.33) and no significant interaction (*t*_349_ = 1.40, *p* = 0.16; figure 2). To further investigate the possibility of an interaction, we ran the same model on the familiar and unfamiliar halves of the dataset separately. Females had a higher lifetime reproductive success when housed with related familiar males than unrelated familiar males, but this effect was marginally non-significant (*t*_184_ = −1.9, *p* = 0.053). However, there was no effect of relatedness when comparing the lifetime reproductive success of females exposed to related unfamiliar and unrelated unfamiliar males (*t*_168_ = −0.123, *p* = 0.90).

There was a significant effect of the interaction between relatedness and day on female reproductive ageing (*t*_895_ = −3.14, *p* = 0.0017), whereby the age-specific offspring production of females housed with unrelated male triplets declined faster than that of females housed with related male triplets. There was no significant interaction between familiarity and day (*t*_895_ = −0.74, *p* = 0.46), nor between relatedness, familiarity and day (*t*_895_ = 1.04, *p* = 0.30), on daily offspring production (electronic supplementary material, figure S2). Again, we ran the model on the familiar and unfamiliar datasets separately. When comparing familiar treatments, the interaction between relatedness and day remained significant (*t*_463_ = −3.23, *p* = 0.0013), with females ageing faster when housed with unrelated triplets. When comparing unfamiliar treatments, there was no significant interaction between relatedness and day (*t*_432_ = −1.53, *p* = 0.13).

**Figure 3.**
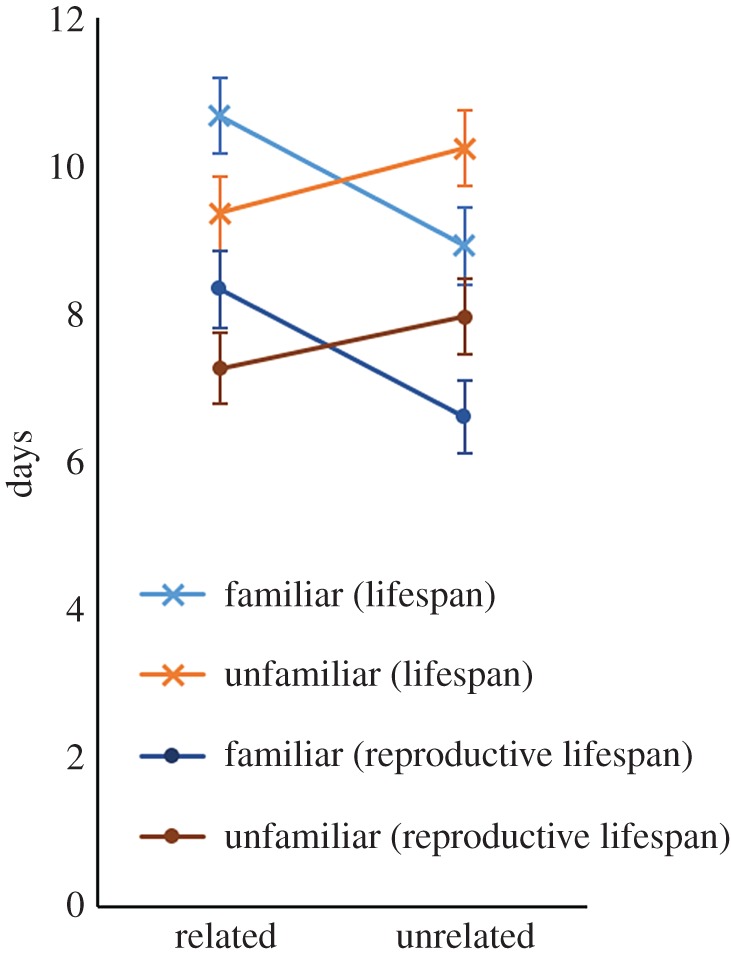
The effect of male relatedness and larval familiarity on female lifespan and reproductive lifespan. The mean number of days from the start of the experiment until the female died (lifespan; crosses) and the mean last day on which the female reproduced (reproductive lifespan; circles), with error bars representing ± one standard error. The last day of reproduction was estimated as the last day of the last time period in which the female reproduced. The interaction between relatedness and familiarity was significant for both lifespan and reproductive lifespan (*p* < 0.05).

There was a significant interaction between relatedness and familiarity on female reproductive lifespan (Wald *χ*^2^ = 5.34, *p* = 0.021; figure 3), whereby females housed with related familiar males reproduced for longer periods than females housed with unrelated familiar males (risk ratio = 1.37, *p* = 0.041; electronic supplementary material, table S5). The interaction between relatedness and familiarity also had a significant effect on female lifespan (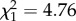, *p* = 0.029; figure 3), with a marginally non-significant trend for females housed with related familiar males to live longer than those housed with unrelated familiar males (risk ratio = 1.322, *p* = 0.069; electronic supplementary material, table S5).

Taken together, these results indicate that female harm is minimized when females are exposed to triplets of males that are both related and familiar to each other.

## 4. Discussion

The role of relatedness in sexual selection and sexual conflict has attracted increasing interest, given the potential for indirect fitness effects in structured populations [6,14,45]. An important challenge in this context is to disentangle the role of relatedness from that of social familiarity. Our results provide support to previous findings from some *D. melanogaster* populations, indicating that related familiar males are less harmful to females. Importantly, the use of a fully factorial design enables the present study to show that both genetic relatedness and familiarity during development are required for any modulation of male harm to females in our population of *D. melanogaster*.

The present study found that females housed with triplets of full brothers show a small but significant increase in reproductive success and slower reproductive ageing than females housed with triplets of unrelated males. While there was no significant interaction between male relatedness and familiarity, this reduction in female harm due to relatedness was only apparent when comparing related and unrelated males in socially familiar treatments. Were familiarity not to play any role in mediating lifetime reproductive success, we would expect to see an effect of relatedness in both the familiar and unfamiliar halves of our data. Therefore, these results suggest that, despite the lack of a significant interaction, familiarity may play a role in mediating the effect of male relatedness on female lifetime reproductive success.

Consistent with this, male relatedness interacted with familiarity to affect both female reproductive lifespan and female lifespan: females both reproduced and survived for longer when housed with related familiar males than with unrelated familiar males, while there was no significant difference attributable to relatedness in the unfamiliar treatments. The role of relatedness can be seen clearly in figure 3: without an effect of an interaction between relatedness and familiarity, we would expect the lines to be flat (no effect of relatedness nor an interaction) or parallel (no interaction). The statistical significance of the effects above was generally weak, and thus some caution should be applied in their interpretation.

Similarly, neither male relatedness nor familiarity affected the rates of male–male aggression, courtship or mating, which seems to contradict previous findings [22,23,26]. The most likely reason for this is that the experimental design of the present study prevented us from replacing the males at regular intervals as in previous studies [22,24–26], and as such, we could not minimize the effects of co-ageing. Male co-ageing with the females is bound to underestimate differences in harm to females across treatments, because males in treatments where they are more harmful to females are also expected to age more quickly (and hence deteriorate faster). Elevated male ageing in high-harm treatments would tend to equalize the levels of female harm across treatments with time, and particularly so towards the end of their lifespan. Thus, our estimates of both overall female harm levels and treatment differences are conservative.

Collectively, these results indicate that, at least in the population we studied, within-group male relatedness plays a role in modulating male harm of females, in consort with familiarity. For all measures of female harm, females experienced the least harm when exposed to males that were both related and familiar. This is consistent with the hypothesis that indirect fitness effects contribute to explain reduced female harm when local male competitors are related to each other. For example, a focal male may be selected to invest less in competition with rival males, and be less harmful to females, if his rivals are more genetically related to him than to the population average and these females are likely to reproduce with his relatives. This is because of the indirect fitness the male would gain via the increased reproductive success of his male relatives, who would experience both less competition for fertilizations and have more fecund mates, and thus gain higher reproductive success. This would reduce both male–male competition and sexual conflict [12]. Males in our study appear capable of discriminating between individuals on the basis on kinship to adopt a less competitive and less harmful strategy with brothers and females, respectively. Crucially, however, we now show that male flies can only do this when raised together as larvae.

These new results help reconcile those of previous studies. Specifically, Carazo *et al.* [22] compared related familiar and unrelated unfamiliar treatments and emphasized the role of male relatedness. Subsequently, Hollis *et al.* [24] added a related unfamiliar treatment, and by showing that related and unrelated males behave the same when unfamiliar to each other, they suggested that harm to females was driven by male–male familiarity. By using a fully factorial experiment, we show that both previous studies capture different aspects of a complex social behaviour: male flies do adjust female harm in response to the relatedness of their rivals, but only under conditions of larval familiarity. The results of the present study therefore also shed light on the proximate mechanisms of kin recognition in *D. melanogaster*. There is some evidence that female *D. melanogaster* preferentially mate with their own relatives [46–48]. In addition, there is evidence that both males and females recognize whether a new partner is related or unrelated to a previous partner (genetic familiarity) [49]. This latter result suggests that kin recognition has the potential to be at least partly based on genetic cues in this species.

Two possible, non-mutually exclusive mechanisms for kin recognition in *D. melanogaster* have been proposed: cuticular hydrocarbons (CHCs) [49,50] and gut microbiota [51]. CHCs have both a genetic and environmental component, and numerous insect species use CHCs to discriminate between kin [52]. Furthermore, CHCs can be modulated by gut microbiota [53], which are maternally transmitted to offspring via the egg and are also strongly influenced by diet [54]. In our study, we separated larvae after 24–36 h, so it is still possible that individuals are using familiarity cues in this very early period of life, which we would detect as an effect of relatedness in this experimental set-up. This would be particularly true if flies were discriminating based on gut microbiota, as these are largely inherited from mothers via the egg casing [55,56]. Our experimental design differs in this respect from previous studies [22–26], which manipulated males at the egg, rather than larval, stage, thus reducing the possibility for maternal cues.

Kin recognition may be costly [57], and both its evolution and maintenance require adaptive explanations, albeit these need not be the same. Our study population has been adapted to laboratory conditions of large, dense, confined populations for over 45 years; over 1000 generations. It is hence possible that this population has not been especially structured beyond the microscale, an unlikely scenario for the evolution of kin recognition.

In this context, two mechanisms may contribute to explain mounting evidence for kin recognition in laboratory-adapted *D. melanogaster* populations: one adaptive and one non-adaptive.

First, these responses may reflect a relic of a plastic behaviour evolved in natural populations. While the initial evolution of this behavioural plasticity would have been presumably costly, the cost of maintaining a plastic response to kin under familiarity may be relatively low in laboratory populations where kin structure is expected to be limited. In this scenario, the evolution of kin recognition mechanisms would have been favoured by persistent population viscosity over multiple generations [25], and natural *D. melanogaster* populations in which recognition would have originally evolved must have been structured such that males could expect to grow up with related and unrelated individuals and compete with familiar individuals as adults.

In the wild, *D. melanogaster* live in orchards, feeding and laying eggs on rotting fruit [58]. Little is known about population viscosity and the family-level genetic structure of wild populations. Females co-locate at oviposition sites [59] (although this is likely due to substrate texture, not females actively seeking other larvae [60,61]), and larvae disperse [62], both of which would potentially reduce the likelihood of stable kin interactions. However, larval foraging behaviour, pupation site and adult choice of resting site all have a strong genetic component [58,63,64], and early adult habitat experiences shape later habitat preferences [65,66]. While there is little information on clutch sizes in wild populations, it is inferred from ovariole anatomy that females lay their eggs in small clutches [67], and indeed laboratory-reared females decide on the site quality between each egg [68]. Small clutch sizes, rather than laying eggs individually, could lead to genetic structure in wild populations by increasing the relatedness among neighbours, increasing the probability that adult males encounter related familiar competitors. There is also evidence of some genetic structure in a wild population, where mating males and females are more related to each other than to the average fly in the population [69].

A particularly important stage for the initial evolution of kin recognition and reduced female harm is likely to be at the colonization of a new patch. If a small number of females initially populate a new feeding site, the next generation will be small and will contain substantial variation in male relatedness. Any behaviours that increase female fecundity and male fitness at this stage of colonization may have large, long-lasting effects on the genetic distribution of the future population. While less relevant in established populations, which are larger and possibly less structured, kin recognition may be retained at relatively low costs as the relic of a highly successful strategy from the founding of the population. Another possibility is that fly populations may show some structure even in laboratory conditions. Flies are known to form non-random social networks even in small group sizes and small physical environments [70], therefore it is possible that some laboratory populations show some degree of relevant microstructure.

The second, non-adaptive mechanism that has recently been put forward to explain kin-biased sexual behaviour in flies is that, if individual levels of competitiveness (e.g. aggression and courtship) are at least partly heritable, triplets of related males are more likely to have similar levels of competitiveness than triplets of unrelated males. If males with similar levels of competitiveness competed less intensely than males with more variable levels of competitiveness, this would produce the effect of related males competing less [26]. This explanation, however, seems counterintuitive. As expected by contest theory and supported by a wealth of data across different taxa, males tend to invest more in competition with rivals of similar competitive value [71,72], a result also replicated by Martin and Long [26]. Also, if female behaviour changes in response to the variability among males, such as being more receptive in the presence of three unrelated (and hence more genetically dissimilar) males [73], this might in turn trigger a proximate increase in male–male fighting and sexual harassment of females, leading to greater female harm.

Another proximate explanation for groups of brothers harming females less than groups of unrelated males might represent a cognitive error. It is possible that if *D. melanogaster* males use variability of smell, be that CHC profiles or gut microbiota, as a measure of how many males they are competing with, they may underestimate the number of competitors when they are related, i.e. smell similar. Thus, if a male is surrounded by brothers, he may assume there is less competition and behave less competitively, harming the female less [13].

While our data show that groups of related familiar males are less harmful to females, we do not yet know whether this is mediated through pre- or post-copulatory effects. It is possible that there are post-copulatory differences between treatments for which we did not test. Male *D. melanogaster* are known to adjust the composition of their ejaculate according to the female's previous mating history and perceived competition [74–76]. In particular, we do not currently know if the levels of male accessory proteins transferred to the females differ between treatments.

The present study joins several others looking at the effect of relatedness on sexual behaviour in *D. melanogaster*, with some of the key findings of each study summarized in electronic supplementary material, table S1. Each used a very similar experimental design, but different laboratory populations of *D. melanogaster*. The Dahomey population used in Carazo *et al*. [22,23] and this study are the same. The three IV populations used in Hollis *et al*. [24], Chippindale *et al*. [25] and Martin & Long [26], while nominally the same, have been reared in separate laboratories for several decades. Apart from genetic differences, the Dahomey and IV populations differ substantially in rearing conditions. The Dahomey population, as used in this study, is maintained in cages with large, dense populations and overlapping generations, which allows for selection to continue late in life. By contrast, the IV populations are maintained on a discrete 14-day generation culture cycle in vials at a controlled density of approximately 100 eggs per vial, which prevents selection from acting beyond that time point. This difference in culturing conditions could potentially alter sexual conflict-mediated selection on female ageing in Dahomey versus IV populations. However, there have been no direct tests of this hypothesis. It will be important for future studies to explore, via the fully factorial design applied here, whether relatedness and familiarity among males similarly interact to affect female harm in the IV and other *Drosophila* populations.

More generally, one implication of these studies is that local relatedness among male competitors may represent a possible modulator of the ‘sexual tragedy of the commons’ and population viability. An important avenue of future research, therefore, will be to explore whether the ecology of *D. melanogaster* across different laboratory and wild populations (e.g. fine-grained population structure) may be more or less conducive to kin-selected sexual cooperation.

## Supplementary Material

Supplementary Tables 1-5 and Supplementary Figures 1-2

## Data accessibility

The datasets supporting this article can be accessed at the Oxford University Research Archive: https://doi.org/10.5287/bodleian:mq112gn2P.
