# Supplementary material for: Male relatedness and familiarity are required to modulate male-induced harm to females in Drosophila
Source: Proc Biol Sci. 2017 Aug 9;284(1860):20170441. doi: 10.1098/rspb.2017.0441 (PMC5563793; doi:10.1098/rspb.2017.0441)

Electronic Supplementary Material for

Male relatedness and familiarity are required to modulate male-induced harm to females in *Drosophila*, 2017, *Proceedings of the Royal Society B*

Sally Le Page[1], Irem Sepil[1], Ewan Flintham[1], Tommaso Pizzari[1], Pau Carazo[2] and Stuart Wigby[1]

[1] Edward Grey Institute, Department of Zoology, University of Oxford, UK.

[2] Behaviour, Ecology and Evolution group, Instituto Cavanilles of Biodiversity and Evolutionary Biology, University of Valencia, Spain.

Supplementary Table 1

Summary of results from previous studies. *The findings from the five previous studies on this topic and the findings from this study on male-male fighting rate, female lifetime reproductive success and female reproductive lifespan. NA means that trait was not observed. The three IV populations, whilst nominally the same, have been reared apart over several decades. The Dahomey populations are all the same population.*

Study	***Fly population***	***Fighting rate***	***Lifetime reproductive success***	***Reproductive lifespan***
Carazo et al 2014 [22]	Dahomey	Lower in related familiar males than unrelated unfamiliar males	Higher in related familiar males than unrelated unfamiliar males	Longer in related familiar males than unrelated unfamiliar males
Hollis et al 2015 [24]	IV (a)	NA	Higher in related familiar males than both related unfamiliar males and unrelated unfamiliar males	No effect
Carazo et al 2015 [23]	Dahomey	Lower in related familiar males than unrelated unfamiliar males	NA	NA
Chippindale et al 2015 [25]	IV (b)	NA	No effect between related familiar males and unrelated unfamiliar males	No effect
Martin et al 2015 [26]	IV (c)	Lower in related familiar males than unrelated unfamiliar males	No effect between related familiar males and unrelated unfamiliar males	No effect
Le Page et al (this study)	Dahomey	No effect	Higher in related familiar males than unrelated familiar males	Longer in related familiar males than unrelated familiar males

Supplementary Table 2

Summary of block main effects and interactions including block. *The experiment occurred over two blocks. When testing each measure of female harm and male behaviour, we included block and all possible interactions of block with the other main effects as a fixed effects. None of these interactions were significant (P<0.05) so were removed from the final model, and block was retained as a main effect. The values below for the interactions are from the full models including interactions, and the values for block as a main effect are from the final models excluding interactions.*

Model	*Effect*	*Result*
Lifetime reproductive success	Relatedness*Block	t_{353}= 0.54, P=0.59
	Familiarity*Block	t_{353}= 1.01, P=0.28
	Relatedness*Familiarity*Block	t_{353}=-0.67, P=0.51
	Block	t_{353}= 4.37, P<0.01
Reproductive Ageing	Relatedness*Block	t_{355}=-0.89, P=0.38
	Familiarity*Block	t_{355}=-0.59, P=0.56
	Relatedness*Familiarity*Block	t_{355}= 0.83, P=0.41
	Relatedness*Day*Block	t_{891}= 1.61, P=0.11
	Familiarity*Day*Block	t_{891}= 1.53, P=0.13
	Relatedness*Familiarity*Day*Block	t_{891}=-1.29, P=0.20
	Block	t_{358}= 4.08, P<0.01
Female lifespan	Relatedness*Block	X^2_1= 0.70, P=0.40
	Familiarity*Block	X^2_1= 0.10, P=0.76
	Relatedness*Familiarity*Block	X^2_1= 0.05, P=0.83
	Block	X^2_1= 8.88, P<0.01
Female reproductive lifespan	Relatedness*Block	X^2_1= 1.02, P=0.31
	Familiarity*Block	X^2_1= 0.07, P=0.79
	Relatedness*Familiarity*Block	X^2_1= 0.39, P=0.53
	Block	X^2_1= 0.55, P=0.46
Male courtship	Relatedness*Block	t_{349}= 0.21, P=0.84
	Familiarity*Block	t_{349}=-0.31, P=0.76
	Relatedness*Familiarity*Block	t_{349}=-0.14, P=0.89
	Block	t_{352}= 2.02, P=0.04
Male aggression	Relatedness*Block	t_{349}= 1.66, P=0.10
	Familiarity*Block	t_{349}= 0.75, P=0.45
	Relatedness*Familiarity*Block	t_{349}=-1.43, P=0.15
	Block	t_{352}= 4.71, P<0.01
Mating	Relatedness*Block	t_{349}= 0.04, P=0.97
	Familiarity*Block	t_{349}=-0.77, P=0.44
	Relatedness*Familiarity*Block	t_{349}= 0.77, P=0.44
	Block	t_{352}=-3.16, P<0.01

Supplementary Table 3

Summary statistics of the frequency of behavioural observations. *Aggression and courtship behaviours were measured as the number of scans per day in which we observed any males performing that behaviour in each vial. Numbers shown are the mean proportion of scans in which that behaviour was shown ± standard error. Mating was measured as whether a mating was observed that day in each vial, with 1 representing mating observed, and 0 representing no mating observed. Numbers shown are the means of these binary values ± standard error.*

Male treatment	*Aggression*	*Courtship*	*Mating*
Related familiar	0.197±0.00788	0.497±0.0133	0.0942±0.0122
Related unfamiliar	0.204±0.00854	0.489±0.0148	0.0764±0.0123
Unrelated familiar	0.201±0.00746	0.534±0.0139	0.0866±0.0125
Unrelated unfamiliar	0.189±0.00759	0.529±0.0134	0.0900±0.01270.0127

Supplementary Table 4

Summary of statistical results from binomial penalised quasi-likelihood GLMM of the frequency of behavioural observations. *Aggression and courtship behaviours were measured as the number of scans per day in which we observed any males performing that behaviour in each vial. Mating was measured as whether a mating was observed that day in each vial.*

Behaviour	*Factor*	*t*	*Degrees of freedom*	*P*
Aggression	Relatedness	0.01	352	0.99
	Familiarity	0.75	352	0.45
	Relatedness*Familiarity	-0.99	352	0.32
Courtship	Relatedness	1.26	352	0.21
	Familiarity	-0.35	352	0.73
	Relatedness*Familiarity	0.45	352	0.65
Mating	Relatedness	-0.01	352	0.99
	Familiarity	-0.78	352	0.43
	Relatedness*Familiarity	0.41	352	0.69

Supplementary Table 5

Risk ratios for female reproductive lifespan and lifespan. *Reproductive lifespan was measured as the last day of the last time period in which the female reproduced. Numbers shown are the risk ratios between male treatments across both blocks with lower and upper 95% intervals in parentheses.*

Ratio	*Reproductive Lifespan*	*P*	*Lifespan*	*P*
Related familiar/Related unfamiliar	0.757 (0.555,1.033)	0.0789	0.750 (0.550, 1.022)	0.0680
Related familiar/Unrelated familiar	0.730 (0.539, 0.988)	0.0414	0.755 (0.558, 1.022)	0.0687
Related familiar/Unrelated unfamiliar	0.912 (0.667, 1.248)	0.563	0.868 (0.637, 1.184)	0.372
Related unfamiliar/Unrelated familiar	0.964 (0.713, 1.301)	0.812	1.007 (0.745, 1.361)	0.962
Related unfamiliar/Unrelated unfamiliar	1.204 (0.881, 1.647)	0.244	1.158 (0.851, 1.576)	0.350
Unrelated familiar/Unrelated unfamiliar	1.249 (0.921, 1.697)	0.153	1.150 (0.850, 1.556)	0.365

Supplementary Figure 1

The effect of male relatedness and larval familiarity on female reproduction over time. *Points show the average number of offspring that reached adult and P13 pupal stage laid each day by each female during the experimental period from the first experimental block (dark points), the second experimental block (light points) and mean daily offspring production for each male treatment across both blocks (crosses, also shown in Supplementary Figure 2). Females mated to triplets of males that were related aged significantly slower than those mated to triplets of unrelated males (P<0.05). There was no difference in reproductive ageing between females mated to triplets of familiar and unfamiliar males (P>0.05)*

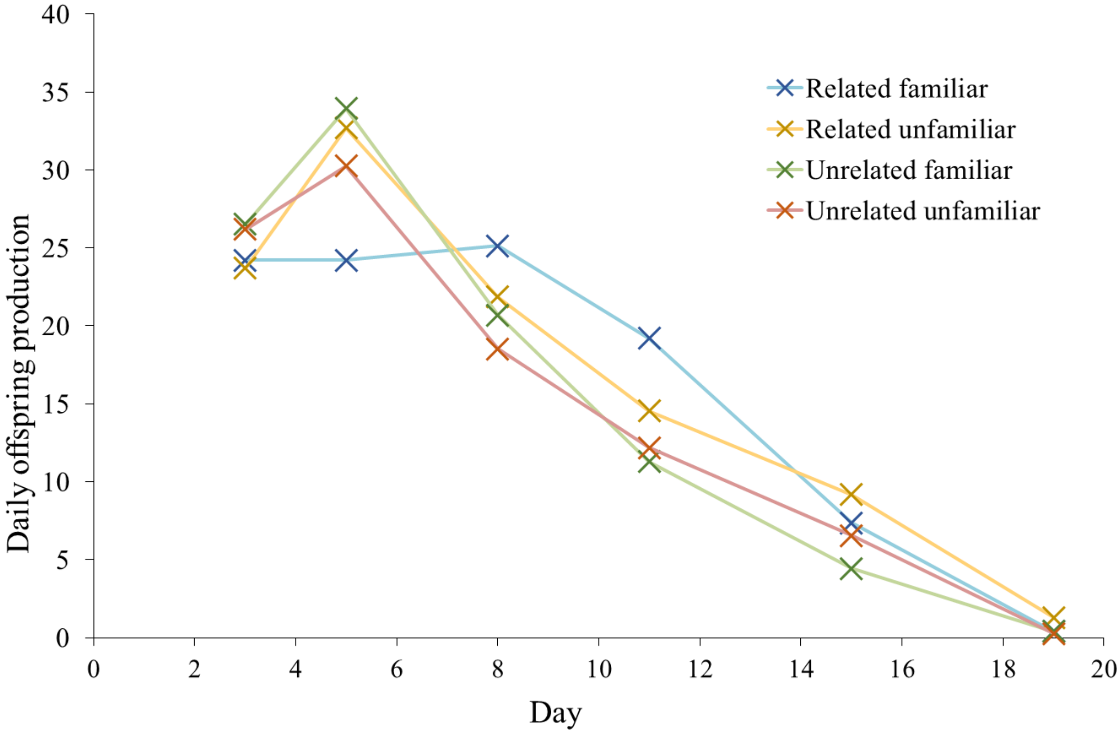

Supplementary Figure 2

The effect of male relatedness and larval familiarity on female reproduction over time. *The mean number of offspring that reached adult and P13 pupal stage laid each day by each female during the experimental period for each male treatment across both blocks. Standard error bars are not shown as the means are an average of two experimental blocks, and the distributions for each treatment and block can be found in the supplementary material. The more negative the gradient of the curve, the faster the reproductive ageing. Females mated to triplets of males that were related aged significantly slower than those mated to triplets of unrelated males (P<0.05). There was no difference in reproductive ageing between females mated to triplets of familiar and unfamiliar males (P>0.05)*

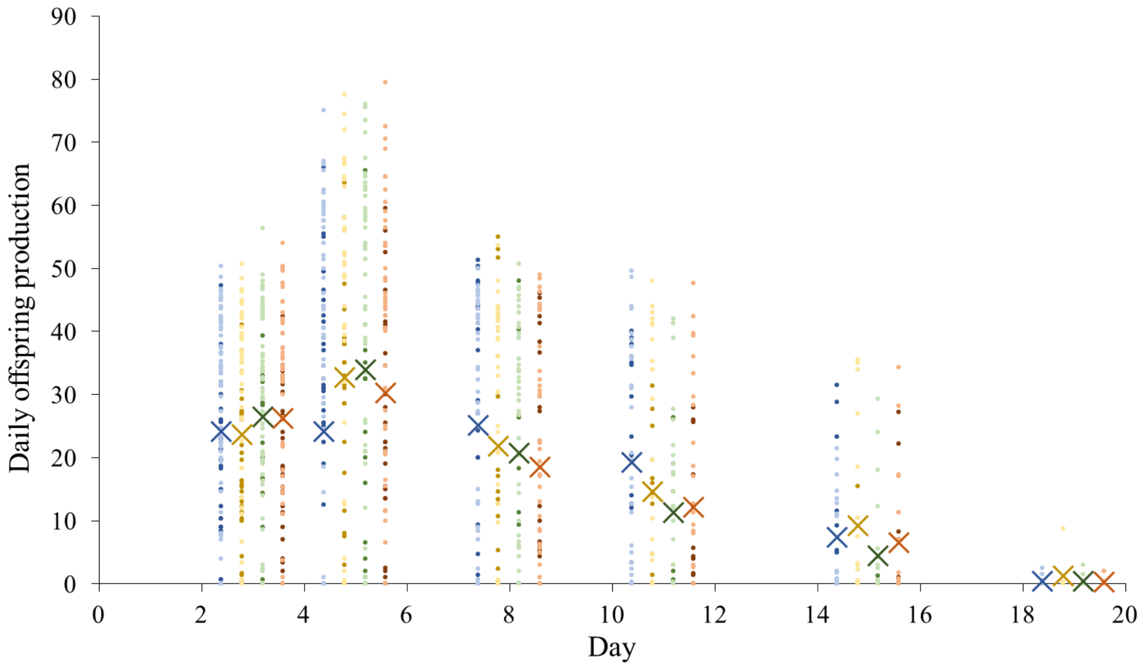

Supplement: Supplementary Tables 1-5 and Supplementary Figures 1-2 [file rspb20170441supp1.pdf]
